# Management strategy of a carotid-esophageal fistula with a right common carotid artery pseudoaneurysm due to chicken bone ingestion: A case report and literature review

**DOI:** 10.3389/fsurg.2023.1129488

**Published:** 2023-04-11

**Authors:** Dongming Xu, Haichao Qian, Lishan Lian, Miaorong Xie, Hanyu Zhang

**Affiliations:** ^1^ Department of Emergency Medicine, Beijing Friendship Hospital, Capital Medical University, Beijing, China; ^2^ General Administration of Customs (Beijing) International Travel Health Care Center, Beijing, China

**Keywords:** common carotid artery pseudoaneurysm, fistula, gastrointestinal bleeding, hemorrhage shock, case report

## Abstract

Common carotid artery (CCA) pseudoaneurysm is a rare clinical disorder. CCA pseudoaneurysm that occurs with a carotid-esophageal fistula and causes massive upper gastrointestinal bleeding is especially uncommon but can be life-threatening. Accurate diagnosis and prompt managements are essential to save lives. Here, we report a case of a 58-year-old female who presented with dysphagia and throat pain after accidental ingestion of a chicken bone. The patient presented with active upper gastrointestinal bleeding which quickly developed into hemorrhage shock. Imaging studies confirmed a diagnosis of right CCA pseudoaneurysm and carotid-esophageal fistula. The patient had a satisfactory recovery after a right CCA balloon occlusion, right CCA pseudoaneurysm excision, and right CCA and esophageal repairs. We present and discuss this case here to remind physicians to rule out rare causes of upper gastrointestinal bleeding. A multidisciplinary approach is commonly required to achieve satisfactory outcomes in these cases.

## Introduction

Carotid artery aneurysm accounts for approximately up to 4% of all peripheral artery aneurysms (1). Carotid artery pseudoaneurysm is the most common type of carotid artery aneurysm (2). The pseudoaneurysm is commonly caused by trauma or endovascular manipulation (3, 4). Most patients are asymptomatic or have pulsating mass in the neck. When a common carotid artery (CCA) pseudoaneurysm has a persistent communication with the esophagus, it can lead to a carotid-esophageal fistula. The affected patient can then present with massive gastrointestinal bleeding and hemorrhagic shock, a life-threatening emergency. Here, we report a case of massive gastrointestinal bleeding and hemorrhagic shock due to the rare causes of a CCA pseudoaneurysm and carotid-esophageal fistula. We share our experience on its diagnosis and treatments in order to improve the understanding and management strategies for this rare clinical scenario.

## Case presentation

**Figure 1:**
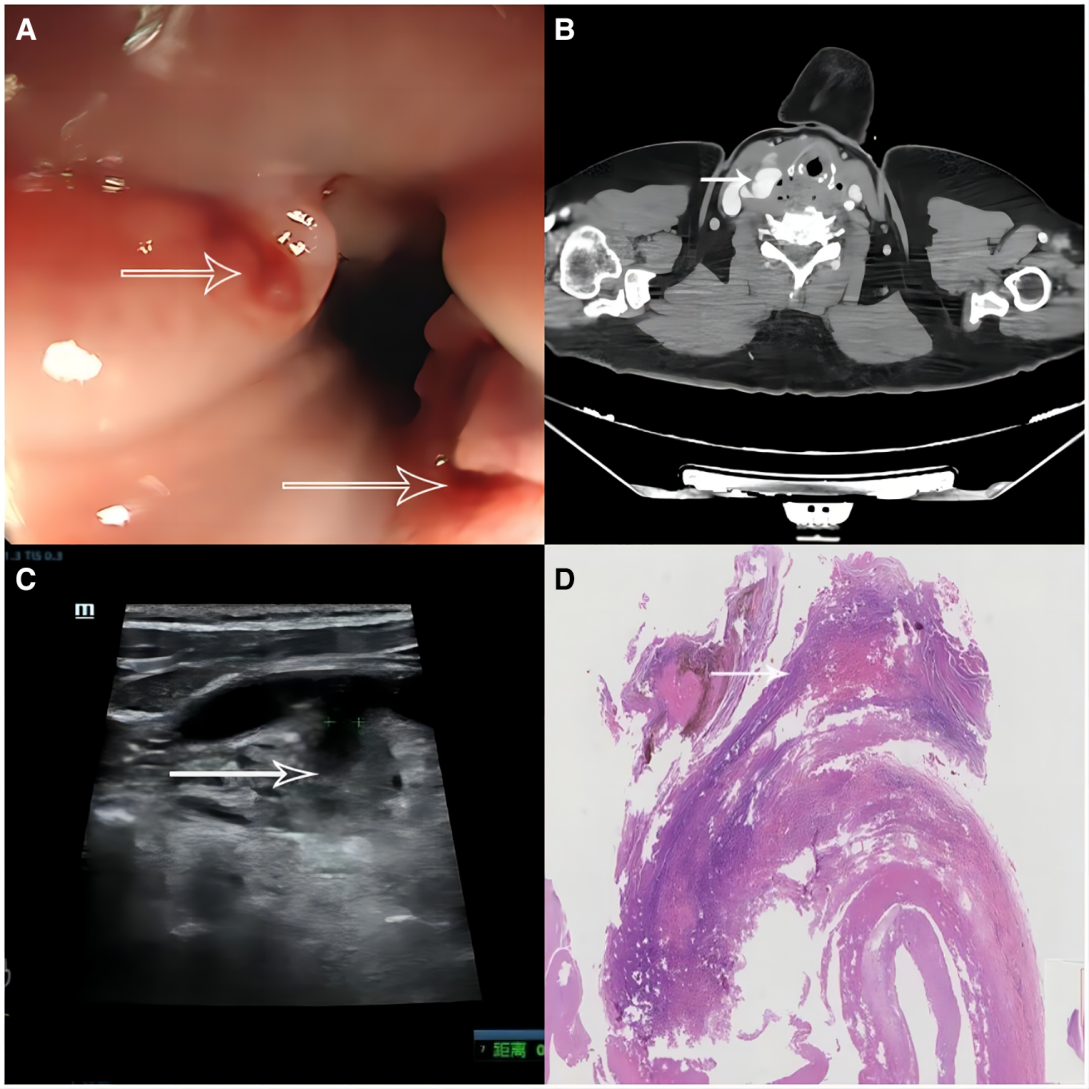
Endoscopic and imaging studies. (**A**) Endoscopic examination revealed two lamellar mucosal lesions in the upper esophagus, located at 11 o'clock and 4 o'clock, respectively (arrows). The mucosal lesion at 4 o'clock was larger and deeper, with dark blood on the surface. (**B**) Contrast enhanced computed tomography scan showed a patchy enhanced shadow extending from the right cervical root to the right common carotid artery (arrow), with surrounding free air bubbles. The shadow was close to the esophageal entrance. (**C**) Ultrasonography examination revealed a 33 × 27 mm cystic common carotid artery pseudoaneurysm with partial thrombosis (arrow). (**D**) Postoperative histopathology report of resected fistula tissue showed infiltration of inflammatory cells (arrow).

On September 25, 2021, a 58-year-old female was transferred to our Emergency Department due to hematemesis and hemorrhage shock. One week prior, she had visited the local hospital with throat pain and foreign body sensation after the accidental ingestion of a chicken bone. An emergent laryngoscopy examination did not reveal any foreign bodies. The patient was discharged but had persistent dysphagia and throat pain. Approximately 10 h prior, the patient started to vomit fresh red blood and returned to the local hospital. An emergent endoscopic examination was performed and revealed two lamellar mucosal lesions in the upper esophagus, located at 11 o'clock and 4 o'clock, respectively. The mucosal lesion at 4 o'clock was larger and deeper, with dark red blood on the surface (Figure 1A). There was also blood in the gastric lumen. Her clinical condition quickly deteriorated, with low blood pressure, tachycardia, and lethargy. Laboratory tests showed a red blood cell count of 4.2 × 10^9^/L, hemoglobin of 121 g/L, and white blood cell count of 15.3 × 10^9^/L. Hemorrhagic shock was considered a risk and as a result she received a red blood cell and plasma transfusion along with dopamine to maintain her blood pressure, before being transferred to our hospital. The patient had a history of hypertension but noncompliance with treatment protocols. The family also reported an allergy to cephalosporin.

At presentation to our hospital, the patient was under an intravenous dopamine infusion. Measurement of her vital signs showed a heart rate of 120 beats/min, respiratory rate of 22 breaths/min, blood pressure of 128/72 mmHg, and temperature of 40.5°C. She looked pale and lethargic. The neck had no mass and the skin was intact. There were coarse breath sounds bilaterally. Heart beats were regular with no murmur. The abdomen was soft, with no tenderness, rebound tenderness, or guarding. Her neurological examination was unremarkable except for lethargy. The laboratory tests showed a red blood cell count of 2.9 × 10^9^/L, hemoglobin of 83 g/L, and a white blood cell count of 26.1 × 10^9^/L. A contrast enhanced computed tomography (CT) scan of the neck showed a patchy enhanced shadow extending from the right cervical root to the right CCA, with surrounding free air bubbles. The shadow was close to the esophageal entrance (Figure 1B). Ultrasonography examination revealed a 33 × 27 mm cystic CCA pseudoaneurysm with partial thrombosis (Figure 1C). In addition, a chest CT scan also showed ground-glass densities, patchy infiltrations, and consolidations in the right middle lobe and bilateral lower lobes. The patient was diagnosed with an upper gastrointestinal hemorrhage, hemorrhagic shock, right CCA pseudoaneurysm, possible right carotid-esophageal fistula, and aspiration pneumonia.

**Figure 2:**
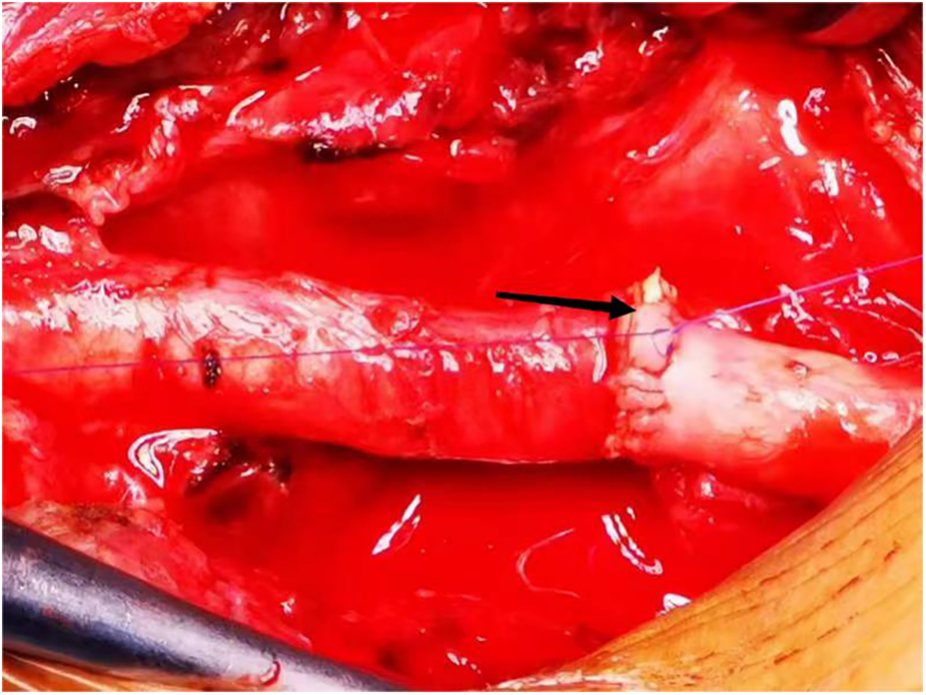
Intraoperative view. The fistula opening in the upper esophagus (arrow).

After presentation to our hospital, the patient still experienced intermittent hematemesis. Intravenous infusions of fluid and dopamine (180 mg + 32 ml normal saline) were continued and tranexamic acid (2,000 mg/day), was also given. Antibiotic treatment with imipenem and cilastatin (1,500 mg/day), were added. Additional red blood cell and plasma transfusions were prepared. Vascular surgery, gastroenterology, and general surgery were consulted. The decision was made to perform an emergency carotid arteriography, which revealed a carotid-esophageal fistula. Under the general anesthesia, the patient received a right CCA balloon occlusion, right CCA pseudoaneurysm excision, right CCA repair with end-to-end anastomosis, and debridement and drainage. The fistula in the upper esophagus was also repaired (Figure 2). During the operation, she received a total of 800 ml red blood cell and 400 ml plasma transfusions. Postoperatively, the patient was admitted into the surgical intensive care unit. The next day, her vital signs gradually returned to normal limits with a heart rate of 76 beats/min, respiratory rate of 14 breaths/min, blood pressure of 141/74 mmHg, and temperature of 36.9°C. Dopamine infusions were stopped. The patient was awake and alert, with no complaints. The surgical site in the neck was clean with no bleeding or signs of infection. The patient gradually progressed from a liquid diet to solid food with good tolerance. Ten days after the surgery, her vital signs were stable. Repeat laboratory tests showed a red blood cell count of 3.0 × 10^9^/L, hemoglobin of 96 g/L, and white blood cell count of 10.7 × 10^9^/L. The histopathology report of resected fistula tissue showed infiltration of inflammatory cells (Figure 1D). Repeat endoscopic examination showed normal esophageal and gastric mucosa with no lesions. Contrast enhanced CT scans also reported normal appearance to the right CCA with a few atherosclerotic plaques. The patient was discharged from the hospital and followed up with in clinic.

## Discussion

Massive gastrointestinal bleeding is a life-threatening emergency (5). Accurately identifying the source of bleeding and immediate hemostatic control are essential to save lives. Common causes of upper gastrointestinal bleeding include peptic ulcers, gastritis, vascular disorders, and tears or inflammation of esophagus (6). Here, we report a rare case of upper gastrointestinal bleeding and hemorrhagic shock due to rare cause of both a right CCA pseudoaneurysm and carotid-esophageal fistula. The patient was successfully treated by CCA balloon occlusion, right CCA pseudoaneurysm excision, right CCA end-to-end anastomosis, and esophageal repair, together with vasopressor support and blood transfusions.

Unlike a true aneurysm that presents with dilatation of all three layers of the arterial wall, a pseudoaneurysm refers to the blood leakage through the vascular wall, forming a hematoma in the surrounding tissue (7). An arterial pseudoaneurysm is commonly caused by arterial injury, endovascular manipulations, or infection (8). Several previous case reports have described patients with CCA pseudoaneurysm resulting from accidental ingestion of foreign bodies, such as fish bones, sewing needles, or metallic wire (9–12). Affected patients presented with a pulsating neck mass and painful swallowing. Artery-esophageal fistula after foreign body ingestion was more frequently reported in aortoesophageal fistula (13, 14). There have only been a few case reports describing carotid-esophageal fistula formation from ingestion of a foreign body (15–17). These patients commonly experienced hematemesis with a more urgent clinical course. In all these cases, a sharp foreign body was identified during the pre-operative imaging examinations and was removed in the surgical exploration. Our patient presented with throat pain and dysphagia after accidentally ingesting a chicken bone ten days before the upper gastrointestinal bleeding began. Although the emergent laryngoscopy examination and CT scan did not reveal any foreign body, an injury from the chicken bone was still a reasonable assumption as the underlying cause of her illness. Although chicken bones are an uncommonly ingested foreign body (18, 19), several studies have reported esophageal, gastric, and intestinal perforations occurring after chicken bone ingestion (20–22). These cases also included reports of aortic pseudoaneurysm and aortoesophageal fistula after ingestion of the chicken bone (14, 23). Our study appears to be the first to report the development of both a CCA pseudoaneurysm and a carotid-esophageal fistula after chicken bone ingestion. Although the chicken bone was dislodged, it may have injured the esophageal wall, which, together with the compression from the adjacent enlarged pseudoaneurysm, could have eventually led to musical necrosis and fistula formation between the pseudoaneurysm and esophageal lumen.

Both ultrasounds and CT scans have a high sensitivity and specificity to diagnose arterial pseudoaneurysms (12). However, detection of a fistula in the esophageal wall may require close inspection during surgical exploration. Once a fistula forms between the carotid artery pseudoaneurysm and the esophagus, patients can present with upper gastrointestinal bleeding. In addition to the supportive care, surgical intervention is usually necessary. Esophageal balloon tamponade, endovascular balloon occlusion, or carotid artery clamping could be applied to immediately control the bleeding. Subsequently, anatomic removal of the pseudoaneurysm and repair of the carotid artery and esophageal wall can be performed (9). A covered stent could also be used to treat a pseudoaneurysm. However, in this case of a pseudoaneurysm in conjunction with a carotid-esophageal fistula, this patient presented a high risk for infection. Thus, we did not place a covered stent to avoid a possible stent infection and recurrent rupture. We chose to perform the pseudoaneurysm excision, end-to-end anastomosis, and debridement and drainage instead. Our patient was successfully managed and had a satisfactory outcome.

Considering previously published cases and our present case, we suggest that several strategies for identifying similar cases. First, physicians should be familiar with various causes of upper gastrointestinal bleeding and record careful medical histories in these patients who present with such bleeding. In addition to common causes such as peptic ulcer and esophageal varices, rare causes, such as esophageal and vascular injury from recent neck trauma, cervical procedures, or foreign body ingestion, should be screened, since these could potentially require emergency surgical repair. Second, physicians should look for the etiology of the upper gastrointestinal bleeding, but at the same time, resuscitate the patient. These patients should be closely monitored and given intravenous fluid infusions, vasopressors, and blood transfusions when necessary. In patients with active bleeding, the decision to provide blood transfusions should not be based on the laboratory measurements of serial hemoglobin levels, but should instead consider dynamic vital sign monitoring, degree of bleeding, estimated blood loss, and the ability to rapidly stop the bleeding. Reversal of anticoagulation might be necessary in patients under anticoagulation treatment. Third, an endoscopy should be performed as early as possible in patients without contraindications (24). An endoscopy can identify the bleeding source but also has therapeutic purposes, such as endoscopic medication injections or band ligation for varices. Fourth, in patients with history of possible foreign body injection, an endoscopy should be performed if the laryngoscopy does not identify any foreign body to avoid missed diagnosis, especially in those patients with persistent symptoms. Fifth, although emergency physicians are the providers who first encounter these patients, a multidisciplinary team approach should be utilized when managing patients with upper gastrointestinal bleeding from a carotid-esophageal fistula. Gastroenterologists and radiologists are important to make an accurate diagnosis, while vascular surgeons and general surgeons are required to perform the surgical repair and intensivists are essential for post-surgical care and resuscitation.

The strength of our present case is that we present the diagnostic and management strategy on a rare case of gastrointestinal bleeding. However, since it was a single case report and various causes could lead to upper gastrointestinal bleeding, this might limit the generalizability of our management approaches. We have discussed our thoughts on similar cases above.

## Conclusion

In conclusion, we report here a rare, but severe, case of upper gastrointestinal bleeding and hemorrhagic shock due to right common carotid artery pseudoaneurysm and right carotid-esophageal fistula, presumably caused by the ingestion of a chicken bone. Physicians should maintain a high degree of suspicion to rule out rare causes of upper gastrointestinal bleeding. A multidisciplinary team approach is commonly required to manage these cases to achieve satisfactory outcomes.

## Data Availability

The raw data supporting the conclusions of this article will be made available by the authors, without undue reservation.
